# The technical expert/clinical user/patient panel (TECUPP): centering patient and family perspectives in patient-reported measure development

**DOI:** 10.1186/s40900-024-00661-4

**Published:** 2025-01-07

**Authors:** Elizabeth Marsolais, Rebecca Anhang Price, Carl T. Berdahl, Anton Shenk, Lucy Schulson, Claire E. O’Hanlon

**Affiliations:** 1 RAND, Santa Monica, CA USA; 2 RAND, Arlington, VA USA; 3 Cedars-Sinai Medical Center, Departments of Medicine and Emergency Medicine, RAND, Los Angeles, CA USA; 4 Section of General Internal Medicine, Boston Medical Center, Boston University Chobanian & Avedisian School of Medicine, Boston, MA USA; 5 RAND, Boston, MA USA

**Keywords:** Patient-reported, Measure development, Technical expert panel, Quality measure, Patient experience, Health care quality, Co-design, Lived experience

## Abstract

Patient and caregiver perspectives are increasingly incorporated into health care research and policymaking, but their inclusion in the quality measure development process often is not robust. We describe a stakeholder panel model for incorporating patient/caregiver voices in the development of patient-reported measures, the Technical Expert/Clinical User/Patient Panel (TECUPP) model. This model is characterized by significant or equal representation of people with lived experience of the disease or condition (as patients or caregivers) to the clinicians and others with technical expertise who typically comprise technical expert panels. We report key design features of the TECUPP model and how we used this model to develop survey-based patient experience measures of timeliness of cancer diagnosis. We describe benefits and challenges of using the TECUPP model and considerations to guide others who might use it as part of developing patient-centered quality measures, based on our experience convening a TECUPP to inform development of a patient-reported measure on timeliness of cancer diagnosis. Benefits include creating space for significant contributions from patients/caregivers and development of a shared understanding of patient experiences and observability of measure domains between clinicians and patients/caregivers. Challenges include time management and managing conversations outside the project scope. Measure development efforts implementing this model should consider recruiting diverse individuals, scheduling short and frequent meetings, enabling participation from all TECUPP members, developing accessible pre-read materials, anchoring meetings with patient stories, and encouraging multiple communication modes. The TECUPP model promotes discussion and understanding by patients/caregivers and clinicians/measure experts helpful for development of survey-based patient-reported measures.

## Abstract (plain language summary)

Patient and caregiver perspectives are often considered in health care research and policymaking, but are not always meaningfully included when developing metrics for assessing whether health care provided is high-quality or low-quality. We describe a new type of stakeholder panel for advising on the creation of surveys that patients or their caregivers complete to help assess care quality. This stakeholder panel model is called the Technical Expert/Clinical User/Patient Panel (TECUPP). A TECUPP includes significant involvement of patients and caregivers in developing patient-reported measures, including multiple people who have or care for someone who has the disease or condition of interest. TECUPPs ideally have an equal number of patients/caregivers to the other members of the TECUPP, who include individuals with clinical or other relevant expertise in research, measurement, or other technical expertise. The TECUPP model helps create space for patients/caregivers to be heard and contribute diverse perspectives. The TECUPP model also helps clinicians and patients/caregivers develop a shared understanding of health care experiences and the types of survey questions that can be answered by patients/caregivers to inform the quality of health care received. This model includes challenges, like time management and managing off-topic conversations. When using a TECUPP, projects should consider the diversity of participants, have frequent but short meetings, and make space for everyone to participate in discussions, ensure written materials are lay-friendly, begin meetings with a patient story, and encourage use of email/chat in addition to spoken communication.

## Background

Patient perspectives are being increasingly sought to inform health care research and policy. Patient voices have been incorporated into the development of community priorities [1], core outcomes [2], frameworks and clinical practice guidelines [3–6], and other research [7–12].

Meaningfully incorporating patients and caregivers into health care quality measure development is recommended by the Centers for Medicare & Medicaid Services (CMS); “public patient involvement” has also been identified as a key element of consensus-based methods, a methodology which relies on the participation of informed individuals to answer questions through an iterative process [13]. A 2022 review of guidelines related to consensus methodology quality reporting noted that “public patient involvement” was an aspect of the methodology that was not regularly addressed [13]. Consensus methodologies, including the Delphi method, utilize thought leader opinions to answer questions in health care [13]. However, best practice guidance for incorporating patient and family voices throughout the quality measure development process is under development. While many measure development efforts seek input from patients and caregivers to prioritize measure content, existing toolkits [14] and resources [15] promoting patient inclusion in quality measure development note that the highly technical nature of some aspects of quality measure development can make it difficult to incorporate perspectives of patients and caregivers [16]. Technical aspects include use of jargon (e.g., “denominator,” “face validity”), application and interpretation of psychometric method and results, and analysis of medical codes and electronic health record data. CMS suggests involving patients and caregivers in the measure development process, through focus groups and Technical Expert Panels (TEP) [15]. In practice, only one or two TEP members are patients or caregivers. Often, these individuals have significant patient advocacy experience and health care domain knowledge which may not be representative of most patients and caregivers.

One way to increase the patient-centeredness of quality measures would simply be to include more patients and caregivers on a measure development TEP. Existing guidance from CMS and measure endorsement organizations includes discussions of patient/caregiver perspectives at multiple stages of measure development [17], including in measure submission documentation to a consensus-based entity [17], public comment periods, TEPs [18], and participation in formal Endorsement & Maintenance Committees [19]. A previous effort pioneered a model to increase the number and involvement of patients/caregivers in quality measure development [20]. This “Technical Expert/Clinical User/Patient Panel” (TECUPP) informed development of patient-reported measures for outpatient palliative care [16, 20]. A TECUPP differs from a TEP in that a TECUPP, by design, includes significant representation of patients and caregivers. The patient and caregiver representation is ideally equal in number to clinical and measurement experts. We have expanded and adapted this model by ensuring equal representation between individuals representing technical and clinical perspectives and individuals with lived experience and by engaging with the TECUPP across multiple meetings for several months.

Integrating patients and caregiver voices is important when developing patient-reported measures, as these measures are designed to reflect patient priorities and are collected directly from patients and caregivers. Patients have a unique perspective on what constitutes quality of care [1, 3, 21], and patient-reported measures are known to correlate with other important aspects of health care quality and health outcomes [22, 23]. There are calls to develop new patient-reported measures from thought leaders in diagnostic excellence who have suggested that patient-reported measures may help to identify failures in the diagnostic process that could inform quality improvement [24, 25].

This paper describes our adaptation of the TECUPP model as part of an effort to create new survey-based patient experience measures of timeliness of cancer diagnosis. The study included an environmental scan (thought leader interviews, literature review, review of existing measures), cognitive interviews on the survey instrument, and a survey pilot test, in addition to the TECUPP; manuscripts describing these other aspects of the study are in preparation. The study, inclusive of the TECUPP, was approved by our institutions’ internal review board. The project convened a TECUPP periodically for a year to solicit input and feedback to inform the patient survey used to collect data for the measure. We describe the design of the TECUPP model and highlight practical and logistical considerations for research teams considering implementing or adapting this model. We conducted a word count analysis to structure the quantification of the relative participation of patients, caregivers, clinicians, and measure experts [26].

## Methods: TECUPP description and overview

A TECUPP is a panel of informed stakeholders, representing lived, clinical, and technical expertise. The key feature of the TECUPP is significant and ideally equal representation of participants with relevant lived experiences to those with clinical or technical expertise. Increasing the number and proportion of patients and caregivers on the panel ensures that the patient/caregiver perspective extends beyond that of a single individual or a patient advocate who may have much higher health system literacy than the average patient.

Incorporating the TECUPP’s feedback may help address face validity concerns in future stages of quality measure development [20]. However, implementing a TECUPP may involve process tradeoffs. While the feedback a TECUPP provides is valuable, extra time and resources may be needed to ensure that meeting materials (e.g., background information, agendas, and meeting summaries) are comprehensible to all TECUPP members and there is time for those with a lived experience to share their stories and perspectives. However, losses in efficiency to the discussion may be offset by gains in its richness.

We convened a TECUPP for our measure development project with the specific overarching goal of developing a shared understanding among patients, caregivers, clinicians, and measurement experts of what should be included in a survey about timeliness of cancer diagnosis. We aimed to develop a survey that was accessible to the patients and caregivers who would complete it and would identify meaningful and actionable information about the quality of care patients received.

### Recruitment

We sought to recruit a diverse set of patients and caregivers with respect to gender, race, ethnicity, age, geography, cancer stage and type, initial presentation type (e.g., abnormal screening, incidental finding, or symptom-driven), diagnosis experiences, and experience with patient advocacy. As certain groups are overrepresented in patient advocacy, we identified potential TECUPP members from a variety of sources. Patients/caregivers were identified using recommendations from thought leaders, previous panels on related subjects, and by contacting individuals who had disclosed having cancer in news or social media. We recruited clinicians who treated patients at various stages of the diagnostic process, including primary care physicians, specialists, and emergency physicians. We also recruited researchers in diagnosis, as well as individuals with technical expertise in quality measurement. Researchers were identified by and from the thought leaders we interviewed, the literature search, suggestions from colleagues, and an open call through the Society of General Internal Medicine listserv. We offered all TECUPP members the same $500 honorarium for a “good-faith effort to participate.”

### Meeting length and frequency

TECUPP meetings were conducted virtually. Virtual meetings allowed us to include participants from diverse geographic areas at low cost. Rather than hosting a one-day or a few half-day meetings, we scheduled six 90 min meetings over a year. The meeting duration was chosen to promote participation by people who might not be able to take a full day away from work or family responsibilities. Convening multiple meetings allowed members to engage throughout the project while also allowing TECUPP members the flexibility to miss occasional meetings as needed.

The six meetings were scheduled at strategic points in the development of the survey. The first four TECUPP meetings were held on a biweekly basis to facilitate rapid iteration on potential domains for inclusion on the initial survey draft. After the fourth meeting, we reflected on TECUPP feedback and iterated internally on the survey draft over the course of a month; this draft survey was discussed at the fifth meeting. The final meeting was a report of the results of the pilot survey and a discussion of reactions; it was held nine months later.

### Pre-meeting preparation materials

We distributed materials to TECUPP members to review a week before each meeting. We considered making two versions of the pre-meeting materials, a layperson version and a version with more technical jargon, but after internal discussions, we decided to create a single version to share with all TECUPP members. This ensured everyone had access to the same project information using the same terms with the hope that would facilitate a balanced discussion. The materials included agendas for the current and all previous meetings, summaries of previous meetings, biographies of TECUPP and measure development team members, information on the project and research ethics, and the draft survey questions.

### Meeting agendas

Each meeting started with a preview of the agenda (Table 1) and a reminder of the established ground rules for participation (Table 2). Then we asked a patient/caregiver member of the TECUPP to share their story of cancer diagnosis. Patient and caregiver narratives emphasized the non-linearity and uncertainties of patients’ diagnostic journeys. Next, we spent approximately 60 min discussing individual survey questions, prioritizing domains, and/or reviewing the findings of the pilot survey. This content was informed by the results of an evidence scan, including literature reviews, and thought leader interviews. The evidence scan aimed to identify similar existing surveys and important domains to cover. Due to the limited time available for TECUPP meetings, we sometimes asked TECUPP members to prioritize items for discussion using in-meeting technology, such as chat and polls. For example, after sharing the initial draft survey with TECUPP members, we used an in-meeting poll to solicit input from TECUPP members on which survey domains were most important. We closed each meeting by reminding participants of next steps and the date and time for the next meeting.

**Table 1:** Sample technical expert/clinical user/patient panel (TECUPP) meeting agenda*

Welcome (5 min)
Patient/caregiver story (20 min)
Discuss potential survey questions (25 min)
Prioritization exercise (35 min)
Wrap-up and next steps (5 min)

*After beginning the meeting with a patient story, the order of agenda items was flexible to respond to TECUPP member priorities

**Table 2:** Ground rules reiterated at start of technical expert/clinical user/patient panel (TECUPP) meetings

Please use plain language
Please turn on your camera if possible
Mute when not speaking
Feel free to unmute and speak
Please use the chat

### Discussion topics

We elicited input from TECUPP members in several areas:

#### Survey domains

We guided TECUPP discussion by laying out pre-established criteria for priority survey content [27]. First, survey items needed to identify aspects of care that were important to patients and caregivers during the cancer pre-diagnosis period for patients and caregivers. Second, they needed to be observable by patients and caregivers. Lastly, they needed to be actionable by health systems, providers, or payers.

#### Survey eligibility

In the TECUPP we discussed timing of the survey (how long after diagnosis patients should receive the survey, and how far back we could expect them to remember events), as well as whether proxies (i.e., informal family caregivers) could be expected to have sufficient information to complete the survey if patients were unable to do so.

#### Survey questions

We discussed which individual questions were most and least important to include and if and what kinds of questions may be missing. In addition, we solicited their proposed refinements to individual draft survey questions and response options.

### Creating an inclusive environment

To create an inclusive environment for discussion, we referred to all TECUPP members by first name rather than titles (e.g., Dr.) and last names. Similarly, we tailored our meeting facilitation to ensure that all TECUPP members were offered opportunities to speak in each meeting by having the meeting facilitator invite individuals who had not yet participated to speak. We emphasized that we would consider feedback offered verbally and the chat equally, which allowed TECUPP members the opportunity to provide input in the manner with which they were most comfortable. We inserted comments and questions typed into the chat into transcripts at the appropriate timepoint by combining the saved meeting chat with the audio transcription of each meeting. We also used the poll feature to collect quantitative information about areas of priority for the survey and to quickly gather input on high-level questions about the importance, relevance, and actionability of different survey domains or questions. In addition to offering multiple ways to provide input during the TECUPP meetings, we welcomed asynchronous feedback from TECUPP members through email or individually scheduled meetings.

## Results

Our TECUPP resulted in notable benefits and challenges for quality measurement development.

### Benefits of the TECUPP model for measure development

#### Patients and caregivers made significant contributions

**Fig. 1:**
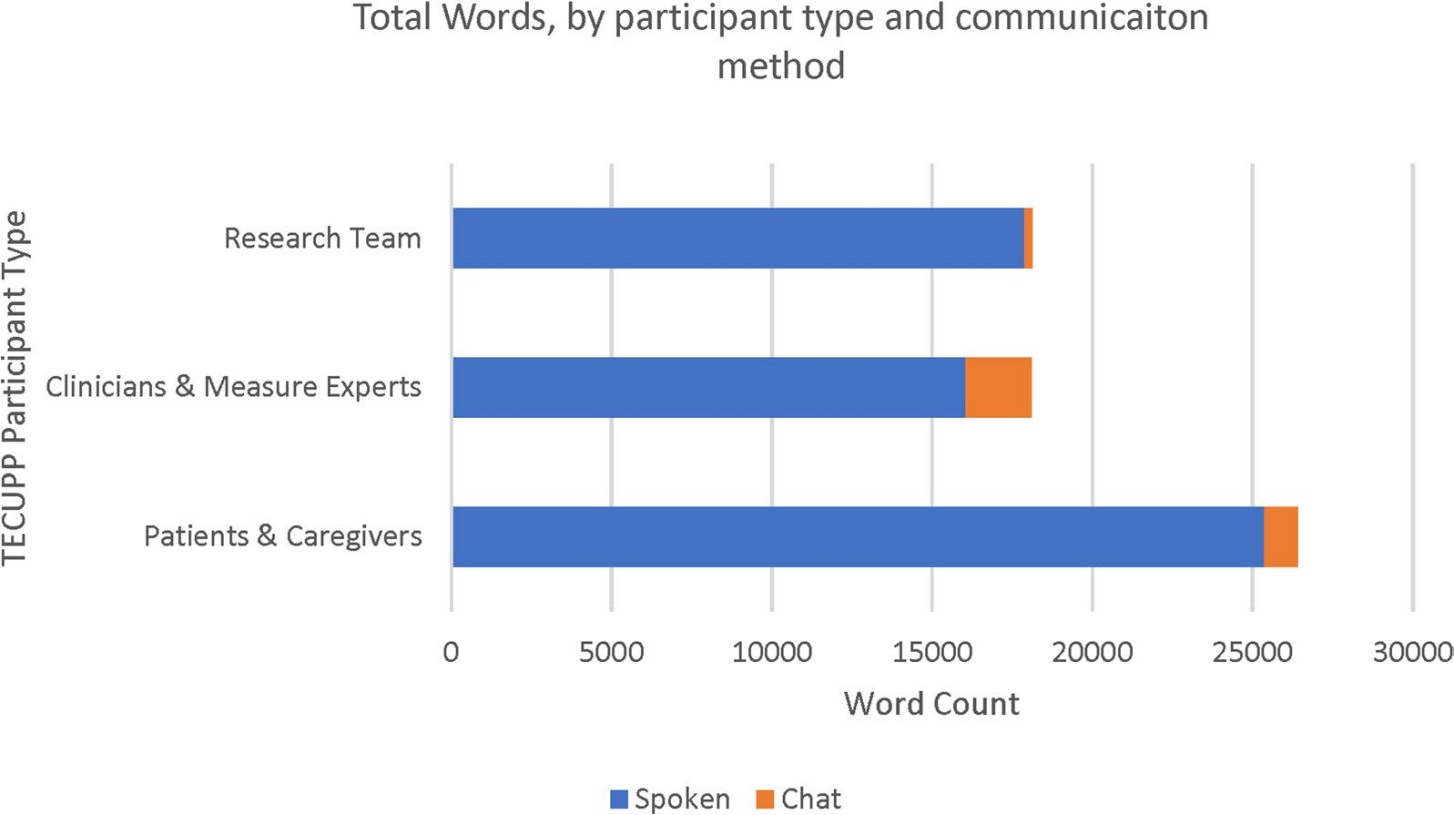
Total number of words contributed during TECUPP meeting discussions by participant type and communication method. Fig. 1 presents the total number of words spoken and typed in the chat during each of the first five TECUPP meetings. At the sixth TECUPP meeting, we reported on the findings of the field test; as our primary goal was reporting our results, we excluded this meeting from the analysis of word counts by participant

Over the course of the TECUPP, patients and caregivers spoke more than clinicians and measure experts, as measured by a word count of TECUPP meeting recordings (Fig. 1) [26]. Our word count includes the recurring “patient/caregiver story” agenda item as part of the total word count for each meeting, which contributed to the large word count for patients and caregivers, but was not the sole cause of the finding that patients and caregivers spoke more than clinicians and measure experts on average across TECUPP meetings. This level of input is likely much higher than is typically achieved on a TEP that has only one or two members who are patients and caregivers.

Use of the chat feature varied widely, with clinicians and measure experts utilizing the chat more than patients and caregivers. Clinicians and measure researchers used the chat feature to share links to references during meetings and share their diagnostic experiences. Patients and caregivers often used the chat to discuss their personal experiences receiving care. Both patients and clinicians used the chat to express thoughts or ask questions related to the spoken content. Overall, the chat accounted for fewer than 10% of the words contributed to each TECUPP meeting (Fig. 1).

#### Meaningful interactions between patients/caregivers and clinicians and measure experts helped develop a shared mental model

Meeting discussions allowed patients, caregivers, measure experts, and clinicians to interact with one another and refine each other’s ideas. For example, direct communication between patients and clinicians provided an opportunity for patients to learn more about how clinical training contributes to situations where patients and clinicians have different expectations. When a patient expressed frustration that it took too long for their clinicians to take their symptoms seriously, a clinician respectfully and effectively communicated the way doctors are trained to make diagnoses and the lengths of time and processes that are considered “normal” from a clinical perspective. On the other hand, patients were able to directly communicate experiences that significantly and negatively deviated from the norm to remind clinicians and measure researchers that patient experiences vary widely. These open and constructive conversations allowed the TECUPP to develop a shared understanding of the diagnostic process between patients, caregivers, clinicians, and measure experts.

### Challenges of TECUPP model for measure development

#### The patient story required significant time

To ground TECUPP discussions in real-world experience and further a commitment to the importance of the work, we allotted 5–10 min at the start of each TECUPP meeting for a patient or caregiver to share their cancer diagnosis story. These stories revealed important nuances and uncertainties in the diagnostic process and helped make discussions more concrete. However, the stories often took longer than the allotted time (up to 20–30 min, including discussion).

#### Conversations often stepped outside the project scope

Occasionally, TECUPP members brought up topics that were out of scope for the focused agenda or needs of our measure development effort. For example, patients and caregivers often raised issues related to the availability and release of test results in patient portals and the importance of health care navigators and peer support.

#### Gaps in TECUPP membership

Despite thinking broadly about the range of potential stakeholders for our measure of timeliness of cancer diagnosis, TECUPP discussions revealed that it may have been useful to have included a payer (e.g., representative from the Centers for Medicare & Medicaid Services or a private health insurer) in our TECUPP.

## Discussion

Through rich interactions between the measure development team, patients, caregivers, clinicians, and measure experts, a TECUPP can provide substantive input that impacts the content and design of a quality measure. For example, in our project, TECUPP input highlighted the need to prioritize communication and respect as a domain on our patient survey. In addition, based on the TECUPP, we developed different questions for primary care and specialty care. The TECUPP also informed our understanding of the level of detail with which patients/caregivers can reasonably be expected to report on dates and time periods for various events related to cancer diagnosis. Table 3 highlights the key design features of a TECUPP that help to facilitate these benefits to quality measure development, including recruitment of members that are balanced between technical experts and patients and caregivers and approaches for promoting robust participation from patients and caregivers in meeting discussions.

**Table 3:** Key design features of technical expert/clinical user/patient panel (TECUPP) model

Recruit members representing groups that will be evaluated by the proposed measure (clinicians or health systems), groups that would use the measure (patients and caregivers), and other thought leaders and key collaborator groups (substantive and measurement experts, payers)
Ensure significant (and ideally equal) representation of participants with relevant lived experiences to those with clinical or technical expertise
Schedule shorter, iterative TECUPP meetings at key points in measure development
Create an inclusive environment and level playing field
Distribute materials in plain language ahead of TECUPP meetings
Anchor the meetings with a patient story
Encourage multiple modes of communication

Anchoring TECUPP meetings with a story from a patient or caregiver can be time-consuming. In our experience, this time investment is worthwhile; we recommend allocating approximately 20–30 min in the agenda to this story and encouraging continued discussion through the chat feature once the allotted time is complete.

Rich TECUPP discussions sometimes meander beyond the scope of the agenda for the particular meeting or project. To keep discussions focused, we recommend that the moderator remind the group of the meeting agenda, thank the TECUPP members for their insights, and invite continued discussion of off-topic items in the chat.

If TECUPP discussions reveal that the panel is missing an important stakeholder, we recommend that the measure development team attempt to address gaps in perspective by conducting targeted literature reviews or interviews with thought leaders, rather than attempting to add members to the TECUPP midway through the project.

## Conclusions

The TECUPP is a promising and innovative model for centering patient and caregiver voices in the development of patient-reported quality measures. The ongoing dialogue between patients, caregivers, clinicians, measure experts, and other technical advisors can yield productive feedback that positively informs the development of new patient-reported measures as well as other important areas for research inquiry. In our project, TECUPP members’ input significantly informed survey development, such as patients’ ability to recall specific dates and different ways to probe for that information, and topics to prioritize in creating patient-reported measures on timeliness of cancer diagnosis. Others considering this model should pay careful consideration to several design considerations, including TECUPP member composition and meetings agendas and cadence. The TECUPP model can provide useful information to inform the early stages of patient-reported measure development by amplifying patient and caregiver voices.

## Data Availability

Data is provided within the manuscript in the Data Supplement Appendix.

## Data supplement

TECUPP meetings were recorded and automatically transcribed by ZoomGov. A member of the research team corrected and de-identified the meeting transcripts. The word counts below were generated using Microsoft Word’s built in word count feature. We analyzed transcripts for the first five TECUPP meetings. At the sixth TECUPP meeting, we reported on the findings of the field test; as our primary goal was not discussion, we excluded this meeting from the analysis of word counts by participant. The patient story is included in the word count because 1) anchoring each meeting on a patient or caregiver story was a deliberate choice by the project team to shape the discussion, and 2) the patient story was regularly discussed throughout the remainder of each TECUPP meeting to demonstrate the applicability of a particular concept or survey question. For purposes of this calculation, the “patient story” word count was operationalized as the words the patient or caregiver used to communicate their story from the beginning of the patient story agenda item until discussion of the next agenda item began. Any further discussion of the patient story throughout the meeting, such as discussing how that story relates to a proposed survey question, is counted as part of the general meeting word count.

**Table:**

Meeting	Patients & caregivers					Clinicians & measure experts			Research team			All Participants (includes research team)	
	Live (includes patient story)	Chat	Patient Story	Total Words (includes patient story)	Total Words (excludes patient story)	Live	Chat	Total Words	Live	Chat	Total Words	Live	Chat
Meeting 1	5639	242	1601	5881	4280	2676	277	2953	3765	5	3770	12,080	524
Meeting 2	7397	272	3013	7669	4656	2969	795	3764	2253	25	2278	12,619	1092
Meeting 3	3230	136	1055	3366	2311	4175	418	4593	4062	212	4274	11,467	766
Meeting 4	5971	112	1265	6083	4818	1424	101	1525	3452	0	3452	10,847	213
Meeting 5	3118	275	423	3393	2970	4806	445	5251	4354	2	4356	12,278	722
Total	25,355	1037	7357	26,392	19,035	16,050	2036	18,086	17,886	244	18,130	59,291	3317

## Abbreviations

TECUPP: Technical expert/clinical user/patient panel
TEP: Technical expert panel
